# New Direction in Antimicrobial Delivery System: Preparation and Applications of Hydrogel Microspheres

**DOI:** 10.3390/pharmaceutics17040529

**Published:** 2025-04-17

**Authors:** Jiapeng Li, Guotao Wei, Yihao Yuan, Ling Wang, Miaohan Qiu, Bo Li, Ruofei Ma, Jiawei Wu, Ziyi Shen

**Affiliations:** 1 Key Laboratory of Resource Biology and Biotechnology in Western China, Ministry of Education, Faculty of Life Science & Medicine, Northwest University, 229 North Taibai Road, Beilin District, Xi’an 710069, China; 2021113215@stumail.nwu.edu.cn (J.L.); 2021113224@stumail.nwu.edu.cn (G.W.); yuanyihao@stumail.nwu.edu.cn (Y.Y.); 2022113167@stumail.nwu.edu.cn (L.W.); libo@nwu.edu.cn (B.L.); 2 College of Integrated Traditional Chinese and Western Medicine, Shaanxi University of Chinese Medicine, No.1 Century Avenue Middle Section, Xianyang 712046, China; 522020104544@email.sntcm.edu.cn; 3 The First Affiliated Hospital of Xi’an Medical University, Xi’an 710077, China; zuolf@nwu.edu.cn

**Keywords:** hydrogel microspheres, antimicrobial, drug release, biomaterials, tissue engineering, polymer

## Abstract

Antimicrobial delivery systems have undergone extensive development, yet conventional carriers still exhibit limitations such as low loading capacity, inadequate controlled release mechanisms, and cytotoxicity. Recent studies have increasingly demonstrated the potential of Hydrogel Microspheres (HMSs) for antimicrobial delivery. These microspheres exhibit small dimensions, high drug-loading capacity, and the ability to achieve deep-targeted delivery, complemented by adjustable physicochemical properties and biocompatibility that create favorable conditions for antimicrobial transportation. This review systematically examines HMS preparation strategies, characteristic properties, transported antimicrobials, and therapeutic applications. Particular emphasis is placed on critical preparation parameters governing HMS performance, especially those influencing drug delivery dynamics. We conclude by addressing current challenges and proposing actionable strategies for material optimization and clinical translation. This work aims to advance HMS-based antimicrobial delivery systems for more effective infection control.

## 1. Introduction

Bacterial infections have become one of the most serious public health threats worldwide [1]. According to the World Health Organization, hundreds of thousands of people die each year from bacterial infections, and this number could rise to millions by 2050 if no effective measures are taken [2,3]. Addressing bacterial infections is therefore critical and could save millions of lives. One common approach to combat these infections is the use of antimicrobials. However, the standalone use of antimicrobials presents significant challenges, including difficulty in achieving precise dosage, high side effects and cytotoxicity, and poor stability [4,5,6]. In contrast, carrier-loaded antimicrobials have emerged as a promising strategy to improve the pharmacokinetics of antimicrobials, enhance their efficacy, minimize side effects, and reduce the risk of developing drug-resistant bacteria [7]. Common antimicrobial carriers include liposomes, hydrogels, and dendrimers, etc., all of which demonstrate significant potential but still face limitations. For instance, liposomes often have low drug-loading capacity, poor stability, and the potential for sudden drug release [8]; natural polymer hydrogels struggle with deep drug delivery, while synthetic polymer hydrogels often have weak mechanical strength [9]; dendrimers, on the other hand, can be cytotoxic and are expensive to produce [10,11]. A comparative analysis of these carriers is summarized in Table 1.

HMSs offer distinct advantages in antimicrobial delivery, effectively addressing the limitations of traditional carriers in terms of drug loading, release control, and biocompatibility. First, HMSs are made from natural or synthetic polymers that exhibit excellent biocompatibility and biodegradability, creating a stable and low-toxicity environment for drug delivery in vivo [12,13,14]. Second, the microsphere morphology of HMSs increases their surface area, enhancing contact with the lesion site or bacteria. This not only facilitates the local action of antimicrobials, but also improves injectability, making delivery to the target site easier through minimally invasive methods in clinical applications [15,16,17]. Furthermore, the “shear-thinning” or “solid-like” rheological properties of HMSs in particulate form allow for injection without the need for additional chemical modifications, enhancing both ease of use and safety [18]. Additionally, the porous structure of HMSs significantly boosts drug loading, and by adjusting the porosity and chemical structure of the hydrogel network, the controlled release of drugs can be achieved. This prolongs the action time of antimicrobials in vivo, addressing the issue of rapid drug clearance seen with traditional injection methods [6,19,20]. More importantly, introducing functional materials (e.g., photosensitizers, magnetic nanoparticles) or bioactive molecules into the hydrogel matrix can further enhance the antimicrobial effects, reducing the emergence of drug-resistant strains [21,22,23]. Therefore, compared to traditional drug delivery systems, HMSs are more competitive in terms of biosafety, drug loading and release control, and targeted delivery, offering a more efficient and precise option for clinical antimicrobial therapy.

This review aims to explore the strategies for preparing HMSs, the properties that influence their applications and performance, and the types of antimicrobials incorporated into HMSs, along with their applications in the antimicrobial field. Finally, we propose potential directions for improving the performance of HMSs. The primary objective of this review is to evaluate critical parameters in the preparation strategy of HMSs for antimicrobial delivery and their multidisciplinary applications, aiming to provide researchers selecting HMSs as antimicrobial carriers with scientifically validated references. This systematic assessment seeks to enhance healthcare professionals’ capacity for infection control optimization while contributing to the advancement of global health outcomes through innovative therapeutic interventions.

## 2. Materials and Methods

The preparation methods and material selection for HMSs are critical to their performance and applications. Additionally, the type of drug loaded onto the microspheres plays a decisive role in their efficacy for antimicrobial applications. Therefore, evaluating the advantages and limitations of various preparation methods (Table 2), as well as the performance of HMSs loaded with different drugs in antimicrobial applications (Table 3), provides valuable guidance for researchers to select appropriate materials and methods.

### 2.1. Preparation Methods for Hydrogel Microspheres (HMSs)

**Figure 1:**
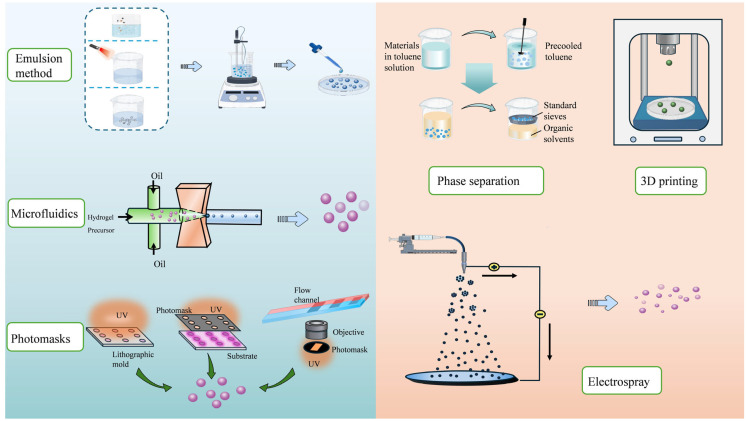
Preparation methods of HMSs. Emulsion methods create particles via dispersed phase solidification. Microfluidics precisely controls droplet formation for uniform particles. Phase separation induces solute separation from the solution. Three-dimensional printing enables layer-by-layer fabrication of complex structures. Electrospraying uses electric fields to aerosolize and solidify droplets. Photomask-assisted patterning allows for precise shape and size control via UV exposure. Each method offers unique advantages for tailoring particle properties for applications in drug delivery, tissue engineering, and more.

A variety of methods have been developed for the preparation of HMSs (Figure 1), with the most common methods including batch emulsion, microfluidics, and spraying methods.

#### 2.1.1. Batch Emulsion Method

The batch emulsion method is widely used for preparing HMSs due to its simple preparation process, suitability for large-scale production, and high cost-effectiveness. This method can be further divided into several subcategories based on the emulsification mode: (1) single emulsion methods, including water-in-oil (W/O) and oil-in-water (O/W) emulsification method; and (2) double emulsion methods, including water-in-oil-in-water (W/O/W) [79,84], oil-in-water-in-oil (O/W/O), and solid-in-oil-in-water (S/O/W) emulsification method. In the conventional preparation process, the hydrogel prepolymer is dissolved in an oil phase containing a crosslinking agent and a surfactant. Stirring then forms a W/O or O/W emulsion [48]. The polymerization reaction is triggered by the combined action of the crosslinking agent and the oil-soluble surfactant, leading to the gradual polymerization of monomers into dimensionally stable polymers. To achieve droplet crosslinking, various techniques, such as ionic crosslinking and photocrosslinking, can be used alongside the introduction of free radical initiators [20,35,58]. It is important to note that the stirring rate and temperature play critical roles during the crosslinking process. A higher stirring rate can cause droplet fragmentation, resulting in smaller particle sizes, while lowering the temperature can promote particle growth and increase their size [85,86,87]. Additionally, some researchers have employed a double crosslinking method to prepare microspheres. Microspheres produced using this method showed excellent morphological stability in both pure water and phosphate-buffered saline (PBS) solutions [20,40].

Although the batch emulsion method demonstrates several advantages in the preparation of HMSs, the resulting microspheres are typically polydisperse, leading to variations between batches [45]. This polydispersity can also result in an uneven distribution of drug loading within individual microspheres. Furthermore, because the process involves the use of an oil phase and surfactants, the HMSs must be thoroughly washed to remove residual oil phase before use. While this washing step is critical, it is also cumbersome and may negatively impact the microspheres’ final performance or the activity of the loaded biofactors if not performed thoroughly [88].

In recent years, inverse emulsion polymerization has gradually developed as a novel method for preparing functionalized microspheres. This technique not only preserves many advantages of traditional emulsion polymerization, but also significantly enhances the physical properties of HMSs [15,48].

#### 2.1.2. Microfluidic Method

As mentioned earlier, achieving fine control over the generation of HMSs is essential to overcoming the limitations of the batch emulsion method [36]. Microfluidics provides an effective solution to this problem. By precisely regulating the microsphere production process, microfluidic technology ensures excellent monodispersity of HMSs [54,89], reduces batch-to-batch variation, and enables the consistent reproduction of similar microspheres [90]. Accelerating the feedstock flow rate decreases the size of the HMSs; however, excessively high flow rates may result in the formation of long tail structures, complicating the separation process [49]. Additionally, increasing the concentration of the feedstock solution also helps enlarge the diameter of the HMSs [54]. It is worth noting that the traditional microfluidic method exhibits relatively low equipment requirements. Furthermore, its precise control over microsphere production significantly reduces defective products, thereby reducing material and disposal costs, which ultimately lowers overall production expenses.

Microfluidics typically involves the introduction of two or more immiscible fluids into a microfluidic device, where shear forces and hydrophobic interactions at the junction of the oil and aqueous phases generate droplets with specific geometries in the oil phase [50]. As a result, the development of microdevices capable of meeting specific droplet formation needs is central to this technology. Commonly used devices in microfluidics include polydimethylsiloxane (PDMS) molds [91] and glass microcapillary devices [92,93].

Although conventional microfluidic methods enable the precise design of HMS structures, they are hindered by time-consuming processes and low throughput [94]. To overcome these limitations, researchers have combined microfluidics with electrospray technology, allowing for the production of hundreds of HMSs per minute. This integrated platform shows significant potential for the efficient, large-scale production of microspheres [53,54]. Most capillary-based microfluidic devices are manually fabricated, making them difficult to adjust or reorganize on demand and limiting their flexibility [89]. This constraint has slowed the advancement of microfluidic technology. In contrast, novel devices utilizing microfluidic chips effectively address these challenges. Microfluidic chip devices offer improved production efficiency and greater design flexibility due to their automation and miniaturization. Although microfluidic chips entail higher initial costs, they enable the integration of multiple production steps onto a single platform, thereby minimizing the need for additional equipment and transfer processes. Moreover, the inherent high-throughput capability and reduced material consumption of the microfluidic method confer long-term cost-effectiveness in continuous production operations [22].

Air microfluidics is an alternative microfluidic method with unique advantages. This method is typically assisted by a nitrogen purge, where the microfluidic device extrudes droplets one at a time, causing large droplets to break into smaller, uniform droplets. These droplets then fall into a collector and undergo crosslinking to form HMSs [20]. In contrast to chip-based microfluidics, air microfluidics supports higher liquid flow rates, leading to a significant increase in particle generation rates. While this method shows great potential for applications, further in-depth research and exploration are needed to fully realize its advantages over other emulsion-based methods and to promote its practical development in the microfluidics field.

#### 2.1.3. Spraying Method

Spraying methods, which include electrospray and spray drying, disperse droplets using high pressure or gas pressure. These methods enable the efficient preparation of microspheres and are well-suited for large-scale production.

Electrospray technology combines a high-voltage power supply, a nozzle, and a liquid feed system [25]. In contrast to the emulsion method, it does not rely on an oil phase and avoids the use of hazardous organic solvents, such as toluene, which can cause environmental pollution [95]. At the same time, electrospray technology allows for the preparation of HMSs at ambient temperature and pressure, effectively preventing the degradation of heat-sensitive bioactives that can occur at elevated temperatures [59].

In this system, a hydrogel precursor solution is delivered to the tip of a needle via a syringe pump, and a voltage is applied. When the applied voltage is sufficient to overcome the surface tension of the liquid, the hydrogel solution splits into micrometer-sized droplets, which are collected in a grounded collector [25,56]. By adjusting key factors such as the applied voltage, polymer flow rate, and needle geometry, HMSs with complex structures in the 1–2 μm size range can be prepared [95]. Therefore, it has enabled a wide range of applications in drug encapsulation and delivery [81]. During the electrospraying process, collecting HMSs by the water bath method can effectively prevent particle aggregation but inevitably causes drug loss. The loss of unencapsulated drug can be mitigated to some extent by shortening the collection time and employing a rapid freeze-drying process [60]. However, when the applied voltage exceeds 10 kV, maintaining the structural integrity of the resulting freeze-dried microspheres becomes challenging [96]. Analogous to the microfluidic chip method, a professional electrospraying apparatus entails higher initial capital investment. However, while requiring greater upfront expenditure, their high-throughput capacity renders them economically viable for long-term manufacturing operations. Notably, the electrospray process typically constitutes a single-step procedure, which significantly reduces temporal expenditures compared with alternative techniques necessitating multiple processing stages and ancillary equipment [25]. Since parameters such as voltage, flow rate, and solution concentration significantly affect the size of the HMSs, operators must have in-depth expertise to achieve optimal results [97].

Compared to electrospray, the spray drying method relies on gas pressure and requires simpler equipment. This method utilizes a compressed gas (e.g., air or nitrogen) to spray a liquid through a nozzle, producing tiny droplets that are subsequently collected [58]. The collected droplets can then be solidified through high-temperature gas drying or crosslinking [61,98]. However, during the high-temperature gas drying process, the elevated temperatures may compromise the activity of the drug [99]. As a result, spray drying should be avoided when working with temperature-sensitive drugs.

#### 2.1.4. Other Methods

In addition to the three common methods mentioned above, other HMS preparation techniques have been extensively researched and applied in recent years, including photolithography, 3D printing, and phase separation, etc. These methods not only offer unique advantages, but also enhance the performance of HMSs in biomedicine, drug-controlled release, and tissue engineering.

In summary, photolithography begins with designing and fabricating a mask containing a microsphere pattern, which is then placed on a substrate containing a hydrogel precursor. The substrate is exposed to UV light, followed by crosslinking and curing. Lithography methods can be classified into three categories: imprint lithography, photolithography, and flow lithography [100]. Imprint lithography utilizes a template with a microstructure pattern and transfers the pattern to the corresponding substrate. The key difference between photolithography and flow lithography is that the former immobilizes a stationary hydrogel precursor, while the latter uses a flowing precursor. The unique advantage of photolithography lies in its ability to achieve nanoscale patterning, enabling the preparation of HMSs with extremely high precision and resolution, a capability that other methods cannot easily match [65]. However, the main challenges associated with photolithography are high cost and low yield. The strategic implementation of reusable photomasks effectively mitigates mold fabrication costs. Concurrently, production scalability can be enhanced through either substrate dimension optimization or deployment of parallel lithography systems, thereby maintaining stable per-unit expenses while significantly boosting throughput capacity.

Three-dimensional printing begins with computer-aided design software to create a three-dimensional model of HMSs. The bioink is then deposited layer-by-layer according to the design using a 3D printer, followed by crosslinking and curing. Compared to other techniques, 3D printing enables the fabrication of HMSs with highly complex internal structures and allows for personalized production, as the design model can be quickly modified, and microspheres can be printed on demand [68]. Song et al. used 3D printing technology to fabricate microspheres loaded with amoxicillin (AMX), which can maintain prolonged drug release and antimicrobial activity, providing a significant sterile environment for bone defect repair [69].

Phase separation is the simplest method for HMS preparation. It primarily involves dissolving the hydrogel precursor in a solvent, then adding a non-solvent or applying low temperatures to induce phase separation, leading to the formation of tiny droplets that subsequently solidify into HMSs. This method is compatible with a wide range of polymers and solvent systems, making it suitable for encapsulating various pharmaceuticals. Cui et al. proposed a biomimetic entanglement-interlocking microphase separation strategy and demonstrated its applicability to various polymerization-induced phase separation materials and hydrogel matrices [71]. However, phase separation has notable drawbacks, including the extensive use of organic solvents, which are difficult to completely remove, and the challenging control of processing conditions.

When selecting a preparation method for HMSs, it is crucial to consider the intended application carefully. For example, batch emulsion is ideal for large-scale production, especially in antimicrobial applications where uniform drug loading and particle size consistency are not essential. On the other hand, microfluidics offers precise control over particle size and release rates, making it an excellent choice for applications requiring continuous drug release and synergistic antimicrobial effects. Meanwhile, spraying is particularly suitable for antimicrobial applications that demand high drug stability and activity, though particle size uniformity is less of a concern. In contrast, photolithography is perfect for light-responsive antimicrobial applications, where precise control over the microsphere shape is necessary to regulate the release of the drug. For even more complex needs, 3D printing allows for the creation of intricate three-dimensional antimicrobial structures and enables the personalized preparation of HMSs. Lastly, phase separation is ideal for antimicrobial applications where particle size uniformity is less critical, but contamination control is a priority.

### 2.2. Materials for the Preparation of Hydrogel Microspheres (HMSs)

#### 2.2.1. Natural Polymers

Natural polymers offer significant advantages in the synthesis of HMSs. Their excellent biocompatibility and biodegradability make them ideal materials for microspheres. Furthermore, the versatility, wide range of sources, low toxicity, and ability to enhance drug stability of natural polymers further enhance their value in drug delivery systems.

**Figure 2:**
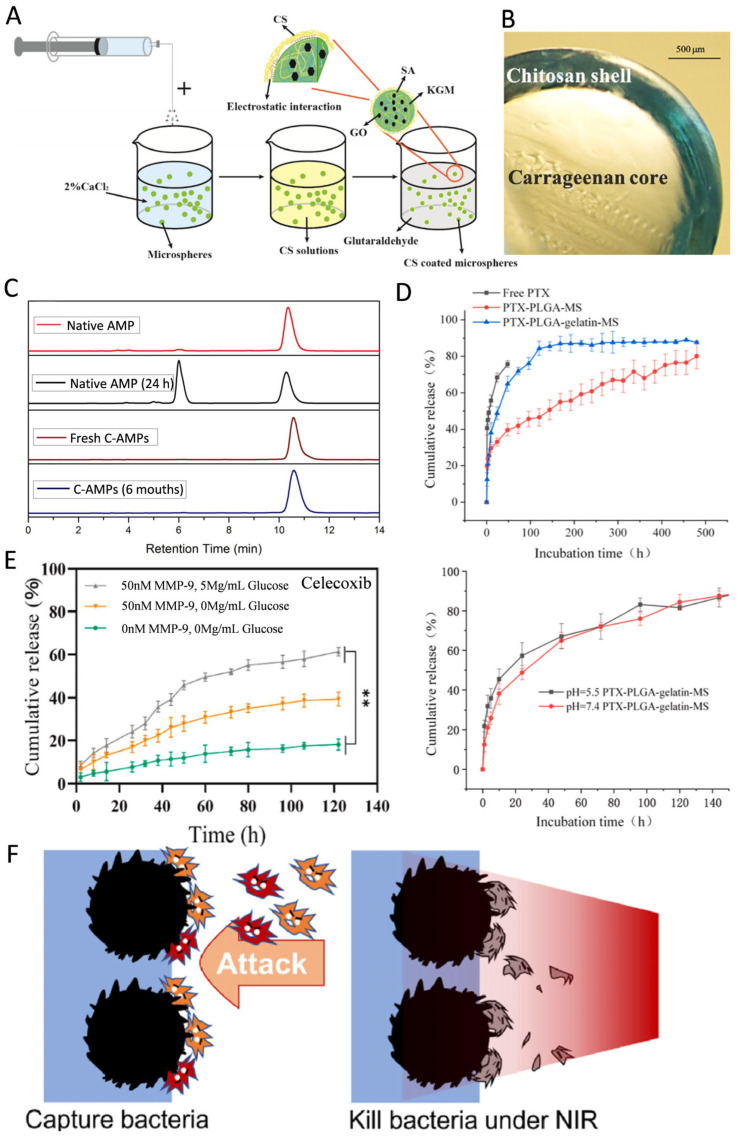
(**A**) Schematic of the formation of the KGM/SA/GO microspheres. Reproduced with permission [96]. Copyright 2019, Elsevier. (**B**) Light microscopy image of the cross-sectioned bead. Reproduced with permission [111]. Copyright 2023, Elsevier. (**C**) Retention time for native Amphotericin B (AMP); native AMP post 24 h in solution; Fresh CS microspheres encapsulating amphotericin B (C-AMPs); C-AMPs after 6 months in solution. Reproduced with permission [31]. Copyright 2018, Elsevier. (**D**) The drug release curves of free Paclitaxel (PTX), smooth and porous PTX-PLGA-gelatin-microsphere. The pH response curve of porous PTX-PLGA-gelatin-microsphere; 144 h after release. Reproduced with permission [112]. Copyright 2023, Elsevier. (**E**) The celecoxib cumulative release of the hydrogels. ** *p* < 0.01. Reproduced with permission [103]. Copyright 2022, Elsevier. (**F**) HMSs trapped bacteria on their surfaces, finally killed them under near-infrared (NIR), thus blocking the attack and protecting the wound. Reproduced with permission [6]. Copyright 2023, Elsevier.

Alginate (Alg), a natural polymeric polysaccharide with low immunogenicity, has been widely utilized in drug delivery systems due to its mild crosslinking mechanism, low cytotoxicity, and cost-effectiveness [13,54,96]. Alg contains anionic carboxyl groups that interact with divalent cations (e.g., Ca^2+^ and Mg^2+^) to form crosslinked microsphere structures. In the microfluidic method, the rapid gelation of Alg with calcium ions can be utilized to minimize the exposure of probiotic bacteria to oil phases enriched with crosslinking agents or surfactants, thereby avoiding damage to the probiotics [50]. Additionally, the presence of carboxyl groups enables Alg to undergo solubilization under neutral and alkaline conditions, protecting drug molecules in acidic environments [101]. As a result, Alg microspheres are particularly well-suited for enteral drug delivery systems, where deprotonation of Alg at intestinal physiological pH triggers microsphere disintegration and releases the encapsulated drug molecules [102]. However, Alg microspheres face limitations such as poor cell adhesion, inadequate mechanical strength, and slow degradation, which restrict their broader use in drug delivery systems [57]. To address these challenges, their properties can be improved through physical crosslinking or by surface coating with chitosan (Figure 2A) [54,96]. For instance, studies have shown that physically crosslinking sodium alginate (SA) with carboxymethyl chitosan and collagen significantly enhances its cell adhesion properties [36].

Chitosan (CS) is a natural biomacromolecule derived from chitin, widely used in the preparation of microspheres due to its excellent biocompatibility, biodegradability, antimicrobial properties, and adhesion [72]. These properties largely stem from the primary amine groups in its molecular structure, which provide reactive sites for the attachment of new functional groups, enhancing the functional versatility of CS. For example, Ma et al. successfully constructed a Schiff base structure by coupling the carbonyl group of citral with the primary amine group of CS, significantly improving its antimicrobial efficacy and reducing the loss of volatile organics in neutral pH environments [35]. CS microspheres also enable controlled drug release, prolonging the duration of drug action [63]. Studies have shown that microspheres prepared from low molecular weight CS encapsulated with antimicrobial peptide can extend drug degradation times to up to six months (Figure 2C) [31]. Additionally, the primary amine groups in CS confer superior adhesion properties compared to Alg, allowing it to firmly bind to a wide variety of biological tissue surfaces. CS possesses inherent antimicrobial properties, showing greater efficacy against Gram-positive bacteria than Gram-negative bacteria [28,43]. This is attributed to its ability to disrupt the integrity of *S. aureus* cells and interfere with cell membrane protein functions, ultimately leading to cell membrane rupture [17]. Furthermore, CS microspheres enhance antimicrobial activity at minimal inhibitory concentrations, reducing the potential toxicity associated with higher doses of antimicrobials [36].

Gelatin, a denatured form of collagen, has become a valuable platform for biomedical applications due to its excellent biocompatibility, biodegradability, non-toxicity, and non-antigenic properties, combined with its cost-effectiveness [22,78]. Gelatin microspheres exhibit high encapsulation rates for water-soluble drugs, making them particularly suitable for these types of drugs [78]. Their encapsulation also effectively prevents the sudden release of drugs (Figure 2D) [34]. Moreover, as a specific substrate for matrix metalloproteinase-9 (MMP-9), gelatin microspheres can degrade in the presence of high concentrations of MMP-9, enabling controlled and accelerated drug release (Figure 2F) [103]. However, gelatin’s low mechanical strength limits its use in applications requiring high mechanical stress. Gelatin methacrylate (GelMA)—produced by the methacrylation of gelatin’s amine groups—has emerged as a successful solution to this issue. GelMA is now widely utilized in HMSs [104,105,106] and in scaffolds loaded with HMSs [58,107], offering enhanced mechanical properties and versatility for various biomedical applications.

Hyaluronic acid (HA) is a naturally occurring acidic mucopolysaccharide found in the human body and serves as a key component of the extracellular matrix [105]. Its high hydrophilicity, biodegradability, and negligible biotoxicity have made HA increasingly popular for drug delivery applications [80,84]. The acetylamino and carboxylic acid groups in HA enable chemical coupling, and modified HA microspheres have demonstrated excellent controlled-release properties for antimicrobial agent delivery [32,33]. These HA-based delivery systems show enhanced antimicrobial efficacy compared to conventional drug delivery vehicles. Additionally, HA can be degraded by hyaluronidase, an enzyme secreted by specific bacteria. This degradation triggers the release of the drug, providing a unique antimicrobial advantage tailored to certain infections [33].

Carboxymethyl cellulose (CMC) is a derivative of natural cellulose obtained through carboxymethylation. Thanks to its biodegradability, biocompatibility, low cost, high water absorption, and non-toxicity, CMC is widely utilized in the development of drug delivery systems [28,73]. The abundance of carboxyl and hydroxyl groups in CMC imparts pH responsiveness to the microspheres prepared from it, allowing the drug release rate to be controlled by adjusting the pH value [34]. To improve the mechanical properties of CMC-based carriers and prevent the burst release of drugs, doping the microspheres with zinc oxide nanoparticles enhances their mechanical strength and coating the surface of CMC microspheres with a layer of CS provides a delayed drug release effect, further optimizing their performance [108].

#### 2.2.2. Synthetic Polymers

Compared to natural materials, synthetic materials offer advantages in terms of superior physicochemical stability, consistency, and ease of manipulation. However, their biocompatibility and biodegradability are often inferior to those of natural polymers, and their processing can be more demanding, which imposes certain limitations on the applications of synthetic polymer microspheres.

Poly (lactic acid-hydroxyacetic acid) (PLGA) is a widely used biodegradable material formed by the co-polymerization of lactic acid (LA) and glycolic acid (GA) monomers [46]. It exhibits excellent mechanical strength and, in biological environments, can be hydrolyzed into LA and GA with minimal side effects. The physicochemical properties of PLGA can be modulated by adjusting factors such as the LA-to-GA ratio, molecular weight, polymer concentration, and the nature of terminal groups. These adjustments directly influence the encapsulation efficiency and drug release kinetics of PLGA-based microspheres [43,60]. However, the inherent hydrophobicity of PLGA limits its clinical applications to some extent. For instance, Chen et al. observed that the hydrophobicity of PLGA microspheres incorporated into a hydrogel reduced the hydrogel’s degradation rate, thereby affecting drug release [109]. Additionally, due to the Hofmeister effect, the LA and GA produced during PLGA hydrolysis may accelerate the degradation of the encapsulating hydrogel, which can further complicate drug release kinetics [60]. As a result, PLGA microspheres require further optimization to address these challenges and enhance their performance.

Polyvinyl alcohol (PVA) is a water-soluble polymer known for its biodegradability, biocompatibility, high solubility, and swelling capacity, making it widely used in the preparation of HMSs [45,79,110]. PVA contains numerous hydrophilic functional groups, which can be combined with silk glycoprotein to improve its degradability, mechanical strength, and antioxidant properties [48].

#### 2.2.3. Composite Materials

When selecting materials for the preparation of HMSs, combining materials with different properties can help compensate for individual material shortcomings and enhance the overall performance of HMSs. For example, the CS/CMC HMS system has been shown to improve the antimicrobial activity of CS under neutral pH conditions while reducing the loss of volatile organic compounds and the oxidation of delivered drugs [27]. This system leverages the electrostatic interactions between CS and CMC to address the poor mechanical properties of CMC carriers and prevent the burst release of drugs [102]. Additionally, multilayer structures formed by polymer shells encapsulating the HMS nuclei can significantly improve microsphere stability and enable more precise controlled drug release (Figure 2B) [38]. For instance, Zhou et al. used polydopamine to encapsulate gelatin microspheres, increasing their surface roughness—a structure beneficial for capturing and killing bacteria (Figure 2F) [6].

Secondary hydrogel-coated microspheres effectively prevent the burst release of drugs and extend their duration of action. In such systems, the inner HMSs may cause an initial burst release of the drug, while the outer hydrogel layer restricts further diffusion, thereby prolonging the drug’s activity [48,78]. Additionally, the hydrogel coating enables the system to firmly adhere to skin wound surfaces, further enhancing the therapeutic efficacy of HMSs [30].

### 2.3. Antimicrobials Delivered by Hydrogel Microspheres (HMSs)

HMSs are capable of effectively delivering a broad spectrum of antimicrobials. These systems load antibiotics, metal nanoparticles, and other antimicrobials through physical encapsulation or chemical bonding. The controlled degradation properties of the microspheres enable sustained, slow drug release, enhancing the bioavailability of the drugs. HMSs not only prevent rapid drug metabolism in vivo, but also minimize side effects on non-target areas, particularly in the treatment of localized infections, where they can precisely control drug release and reduce systemic side effects.

#### 2.3.1. Antibiotics

**Figure 3:**
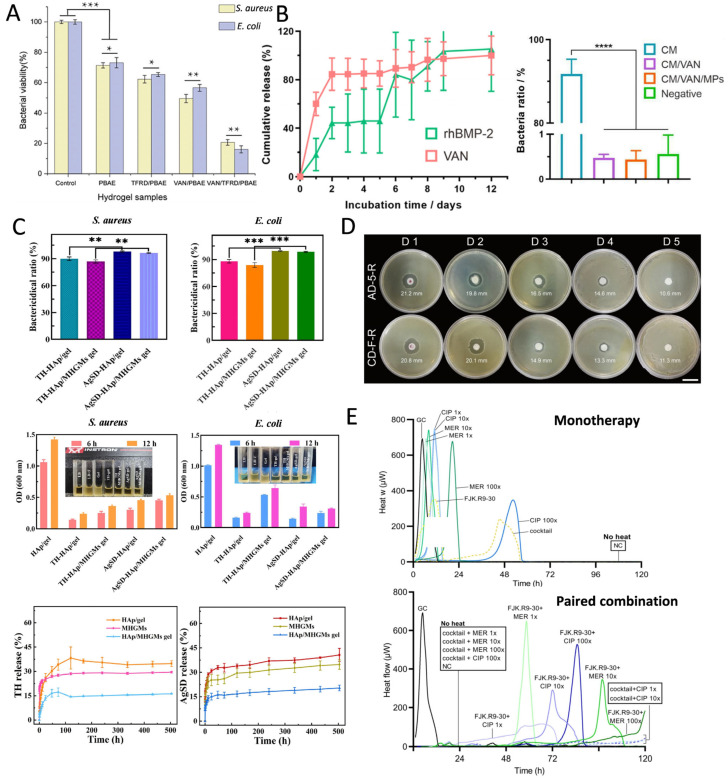
(**A**) Bacterial activity of *S. aureus* and *Escherichia coli* (*E. coli*) cultured in HMS samples for 24 h. (* *p* < 0.05, ** *p* < 0.01, *** *p* < 0.001). Reproduced with permission [29]. Copyright 2023, American Chemical Society. (**B**) The cumulative release of recombinant human bone morphogenetic protein-2 (rhBMP-2) and vancomycin (VAN) of CM/VAN and CM/VAN/MPs films for 12 d. The symbol * represents the significant difference (*p* < 0.05). Quantitative analysis of bacterial ratios in three hydrogel films and negative control to positive control. The symbol **** represents the significant difference (*p* < 0.0001). Reproduced with permission [44]. Copyright 2021, Elsevier. (**C**) The bactericidal efficiency of the hydrogels against *S. aureus* and *E. coli*. OD values (600 nm) of *S. aureus* and *E. coli* suspensions with hydrogels after incubation for a certain time. Cumulative release of tetracycline hydrochloride (TH) and silver sulfadiazine (AgSD) from hydroxyapatite (HAp)/gel. Magnetic hydroxyapatite/gelatin microspheres (MHGMs) and HAp/MHGMs gel in PBS at 37 °C. ** *p* < 0.01; *** *p* < 0.005. Reproduced with permission [78]. Copyright 2022, Elsevier. (**D**) Evaluation of sustained antimicrobial activity of microspheres using the disk diffusion method. The diameter of inhibition zone is marked on the image. Reproduced with permission [49]. Copyright 2019, American Chemical Society. (**E**) Biofilm exposed to phage cocktail as monotherapy. Combinatorial effect displayed by phage cocktail and meropenem/ciprofloxacin. Phage cocktail combined with meropenem completely inhibited biofilm activity (120 h without caloric recovery), significantly superior to monotherapy. Reproduced with permission [57]. Copyright 2023, Elsevier.

Antibiotics are a class of chemical substances that either kill or inhibit the growth of microorganisms and are extensively used in the prevention and treatment of infectious diseases [29]. They can be derived from microbial metabolites (e.g., penicillin) or synthesized chemically (e.g., quinolones). Based on their mechanisms of action, antibiotics are classified into bactericide and bacteriostat. Bactericide directly kill bacteria, whereas bacteriostat control infections indirectly by inhibiting bacterial growth and reproduction. HMSs can deliver a wide range of antibiotics, such as AMX [34], doxycycline [60], and gentamicin [45], among others. The mechanisms of action of antibiotics include the inhibition of cell wall synthesis, nucleic acid synthesis, and disruption of cell membrane function. Therefore, HMSs loaded with antibiotics exert long-lasting inhibitory effects on various pathogenic bacteria (Figure 3). The cefixime-loaded sustained-release composite microspheres demonstrated superior antimicrobial efficacy in experimental trials against *Salmonella typhi* (*S. typhi*) and *Bacillus subtilis* (*B. subtilis*), exhibiting a minimum 10% enlargement in inhibition zone diameter compared with conventional cefixime formulations [83]. Additionally, a study encapsulated rifamycin in alginate/calcium carbonate composite microspheres, which sustainably inhibited *Staphylococcus aureus* (*S. aureus*) for more than five days (Figure 3D), exhibiting prolonged antimicrobial properties [49]. HMSs demonstrate synergistic antimicrobial efficacy through phage-antibiotic co-delivery, achieving a 6.5-fold reduction in bacterial load compared to control groups. Notably, while the conventional administration of antibiotics with free phages induced complete resistance in a subset of bacterial populations, HMS-mediated phage delivery maintained phage susceptibility in 83% of pathogens, thereby effectively suppressing resistance development (Figure 3E) [57].

#### 2.3.2. Metal-Based Antimicrobials

**Figure 4:**
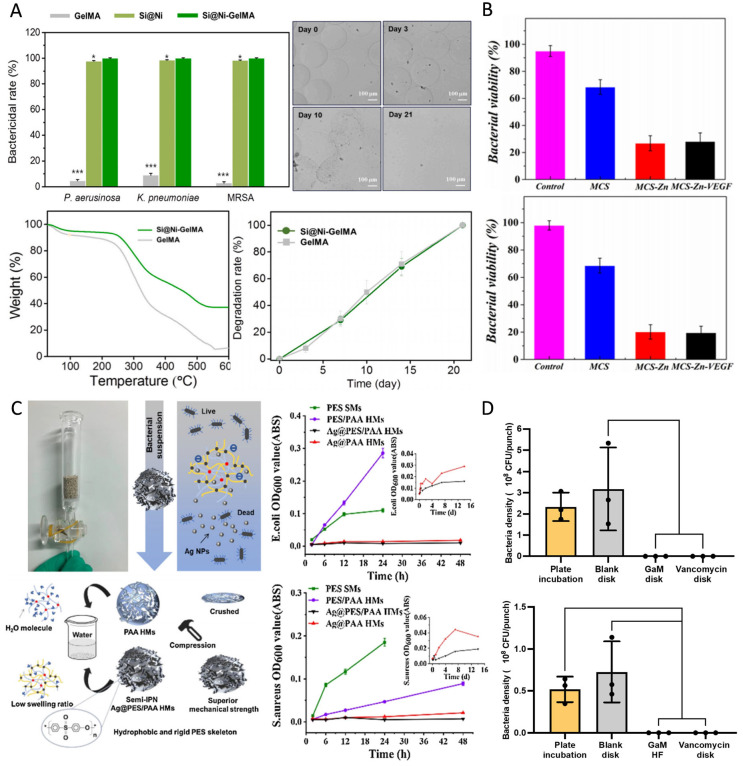
(**A**) The bactericidal rates of GelMA, Si@Ni, and Si@Ni-GelMA against *P. aeruginosa*, *K. pneumonia*, and methicillin-resistant *Staphylococcus aureus* (MRSA). Si@Ni, silicon-based nickel nanoflowers (n = 3). * *p* < 0.05, and *** *p* < 0.001. Bright-field images showing morphological changes in GelMA microspheres over 3 weeks. Thermogravimetric analysis curves for Si@Ni-GelMA and GelMA. Enzymatic degradation of Si@Ni-GelMA and GelMA with 10 IU⋅mL^−1^ type II collagenase (n = 3). Reproduced with permission [51]. Copyright 2024, Elsevier. (**B**) Viability quantitative analysis of *E. coli* and *S. aureus* after co-culture with different microspheres for 24 h. The control group was treated with PBS. Reproduced with permission [53]. Copyright 2022, Elsevier. (**C**) Schematic diagram of the antibacterial column. Schematic diagram of Ag@PES/PAA HMSs with superior dimensional stability. Bacterial growth curves of *E. coli* and *S. aureus*. Reproduced with permission [81]. Copyright 2019, Elsevier. (**D**) In vitro evaluation of GaM-loaded HMS. Zone of inhibition test results after different treatments with bacterial counts per 8 mm punch on different bacterial strains (MSSA 29213 and MRSA 43300). Reproduced with permission [59]. Copyright 2024, Elsevier.

Metal-based antimicrobials delivered by HMSs are widely used due to their broad-spectrum antimicrobial activity and lower susceptibility to resistance (Figure 4). Silver nanoparticles (AgNPs) are among the most commonly used metal-based antimicrobials, primarily exerting their antimicrobial effects by disrupting respiratory enzyme activity and inhibiting the release of adenosine triphosphate (ATP) in bacteria [113]. Gram-negative bacteria exhibit greater sensitivity to AgNPs than Gram-positive bacteria because the thicker, negatively charged cell walls of Gram-positive bacteria hinder the penetration of silver ions. In contrast, the lipopolysaccharides in Gram-negative bacteria enhance AgNPs adhesion, thereby increasing their susceptibility [17]. Xu et al. engineered adsorption columns utilizing AgNPs-loaded HMSs for waterborne toxin removal, achieving complete bacterial eradication (100% bactericidal efficiency). Although these composite systems demonstrated higher minimum inhibitory concentration (MIC) values compared to discrete silver nanoparticles, the incorporated sustained-release mechanism enabled prolonged antimicrobial activity. Significantly, the cumulative silver release was reduced, thereby mitigating environmental contamination risks associated with conventional antimicrobials (Figure 4C) [81]. Additionally, HMSs loaded with AgNPs have demonstrated antimicrobial activity comparable to that of antibiotics [75]. The combined application of metal-based antimicrobials and antibiotics exhibits synergistic antimicrobial effects, reducing both drug dosage and side effects [33]. Beyond AgNPs, other metal-based antimicrobials such as nickel [51], copper [52], gallium (Figure 4D) [59], and zinc [16] have been widely utilized in HMSs for antimicrobial applications. The incorporation of metal and metal oxide nanoparticles not only enhances the antimicrobial efficacy of HMSs, but also improves their mechanical properties [53,81].

#### 2.3.3. Natural Materials

**Figure 5:**
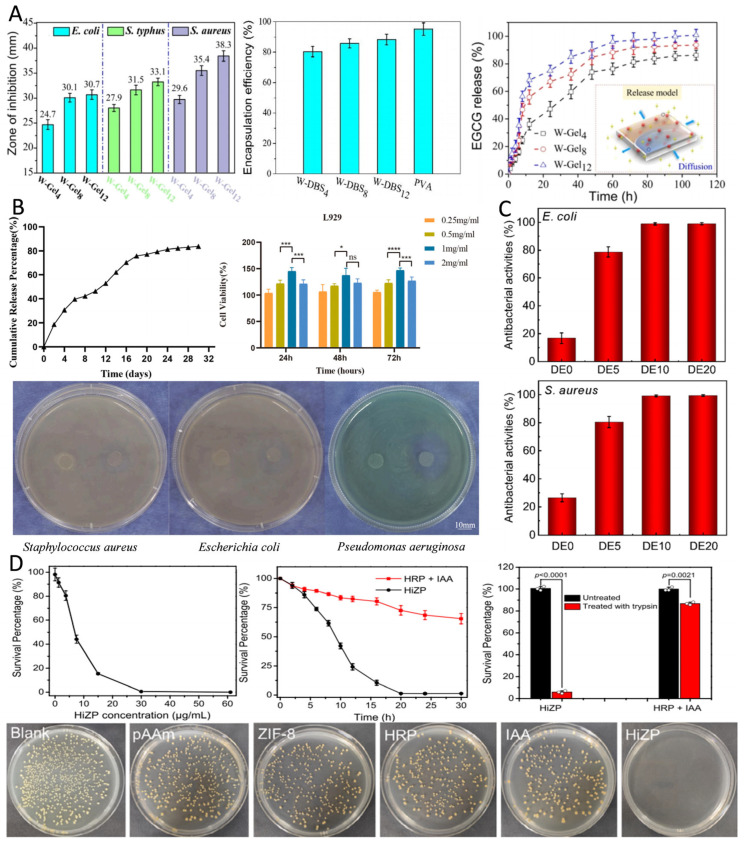
(**A**) Bacterial growth inhibition at pH of 7.4 due to the release of EGCG from HMSs. Encapsulation efficiency of EGCG loaded onto single-network gels at 25 °C for 12 h. Release profiles of EGCG from the double-crosslinked nanocomposite gels (W-Gels) in PBS buffer (pH of 7.4). Reproduced with permission [114]. Copyright 2020, American Chemical Society. (**B**) Cumulative release curve of PLGA@PHMB microspheres. Biocompatibility of cells and microsphere-loaded dermal scaffolds. (* *p* < 0.05, *** *p* < 0.001, **** *p* < 0.0001). ns, not significant (*p* > 0.05). Inhibitory effect of microsphere-loaded dermal scaffolds on different bacteria. Reproduced with permission [46]. Copyright 2024, Elsevier. (**C**) Antibacterial activities of gelatin-based HMSs against *E. coli* and *S. aureus*. Reproduced with permission [74]. Copyright 2022, American Chemical Society. (**D**) Viability of *S. aureus* treated with different concentrations of HRP&IAA@framework-8@polyacrylamide (HiZP). Images of *S. aureus* colonies formed on LB agar plates after different treatments. Time-dependent viability of *S. aureus* treated with the free HRP/IAA system and HiZP. The antimicrobial activities of HiZP and the free HRP/IAA system against *S. aureus* before and after treatment with excess trypsin. Data are presented as mean values ± SD. HiZP, HRP&IAA@ZIF-8@pAAm. Reproduced with permission [55]. Copyright 2022, Elsevier.

Natural materials, such as tea tree oil, citral, horseradish peroxidase (HRP), and indole-3-acetic acid (IAA), have demonstrated efficacy in enhancing bioavailability and exhibiting broad-spectrum antimicrobial activity against a variety of pathogenic bacteria when delivered via HMSs (Figure 5) [35,36,55]. Studies have shown that debranched starch microspheres encapsulating epigallocatechin gallate (EGCG) and loaded in polyvinyl alcohol gels exhibit significant antimicrobial activity against *S. aureus*, *E. coli*, and *Salmonella typhimurium* (*S. typhimurium*) (Figure 5A) [40]. To address the poor aqueous solubility and consequent low bioavailability of mangiferin, researchers developed chitosan-based microsphere encapsulation systems. This formulation leverages sustained-release characteristics to reduce the required drug dosage for equivalent bacteriostatic efficacy while maintaining elevated local drug concentrations, thereby significantly enhancing antimicrobial activity against *Vibrio parahaemolyticus* (*V. parahaemolyticus*) and *Vibrio cholerae* (*V. cholerae*)—a critical advancement for mitigating acute seafood-borne diarrheal diseases [38]. Synthetic antimicrobial materials, such as polyhexamethylene biguanide (PHMB), which exhibit broad-spectrum antimicrobial properties and low biotoxicity, achieve controlled release through HMS encapsulation. These systems demonstrate sustained bacterial suppression throughout 12-day incubation periods compared to free PHMB solutions (Figure 5B) [46].

#### 2.3.4. Other Antimicrobials

In addition to delivering the several common antimicrobials mentioned above, HMSs have been successfully applied to the delivery of various other antimicrobials, including antimicrobial peptides, lysozymes, and probiotics. They have even been innovatively used to deliver *Chlamydomonas reinhardtii*, each of which has shown excellent results in enhancing antimicrobial efficacy and addressing specific challenges.

The delivery of probiotics within the human body is a reliable antimicrobial strategy. However, the main challenge is the lack of delivery systems that can maintain probiotic activity over extended periods and are compatible with the microenvironment [115]. On the other hand, using HMSs for probiotic delivery shows promising potential. Recent investigations demonstrate that Alg microsphere-encapsulated *Lactobacillus reuteri* (*L. reuteri*) hydrogel dressings effectively overcome the limitations of low probiotic viability and suboptimal colonization efficiency observed with free bacterial suspensions. Antimicrobial assessments revealed remarkable pathogen eradication rates exceeding 90% against *S. aureus* and *E. coli*, representing greater bactericidal efficiency compared to non-encapsulated *L. reuteri* formulations [50]. Further research successfully encapsulated active *L. reuteri* in HMSs using a covalent crosslinking technique, demonstrating significant antimicrobial effects against *S. aureus* [32].

Antimicrobial peptides, such as ε-polylysine, delivered via HMSs have demonstrated significant antimicrobial effects, effectively inhibiting *E. coli*, *S. aureus*, and *Pseudomonas aeruginosa* (*P. aeruginosa*) [15,74]. The strategic encapsulation employing the inherent bacteriostatic properties of CS synergistically enhanced antimicrobial efficacy, demonstrating a 5.3-fold increase in bactericidal activity compared to standalone antimicrobial peptide applications [27]. Additionally, the antimicrobial efficacy of lysozyme, a natural antimicrobial enzyme, was significantly enhanced within the HMSs delivery system. Chen et al. fabricated lysozyme-MXene co-encapsulated SA microspheres via microfluidic technology. Under NIR irradiation, these composite microspheres exhibited rapid photothermal response, enhancing lysozyme’s antimicrobial efficacy from 37.5% to over 98% through thermally augmented enzymatic activity [24].

*Chlamydomonas reinhardtii* presents an innovative therapeutic strategy for anaerobic infections. Jun Kang et al. designed an SA microsphere delivery system that harnesses *Chlamydomonas reinhardtii* ’s ability to photosynthesize under light exposure, thereby improving intracellular oxygenation while inhibiting anaerobic bacterial survival. This approach opens new avenues for treating anaerobic infections [80].

## 3. Properties Affecting the Application of Hydrogel Microspheres (HMSs)

**Figure 6:**
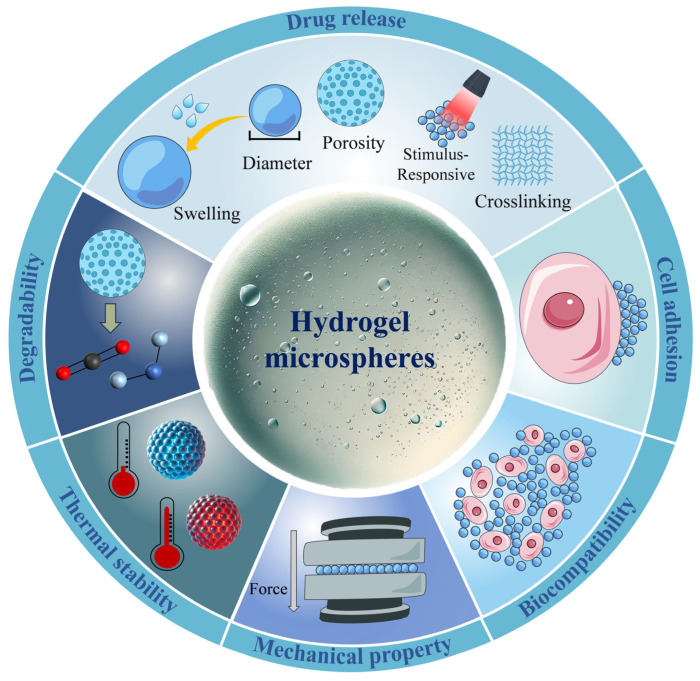
The properties influencing the applications of HMSs mainly include drug release, cell adhesion, biocompatibility, mechanical performance, thermal stability, and degradability. Among these, swelling degree, diameter, porosity, stimulus-responsiveness, and crosslinking degree further affect the drug release process. Precise control over these parameters is crucial for tailoring HMSs to specific therapeutic needs.

HMSs have diverse applications in tissue engineering, regenerative medicine, targeted drug delivery, and environmental protection, among others. Several factors influence their effectiveness, including preparation methods, material selection, and intrinsic properties of the HMSs such as drug release behavior, biocompatibility, cytotoxicity, and mechanical strength. For instance, in filtration and adsorption applications, HMSs must exhibit sufficient mechanical strength to endure water flow pressure and impact [62]. In contrast, when used for in vivo drug delivery, HMSs must remain stable at body temperature to prevent premature drug release caused by temperature fluctuations [45]. By fine-tuning these properties, HMSs can be tailored for a wide range of applications. The properties affecting the performance of HMSs are summarized in Figure 6.

### 3.1. Drug Release

Inappropriate drug release can significantly impact drug efficacy, leading to reduced therapeutic effects and drug wastage. For HMSs, tuning parameters such as diameter, porosity, response release, and targeted release can modify drug release behavior to achieve the desired release profile.

#### 3.1.1. Diameter

Among these parameters, particle diameter is the most critical factor influencing drug release behavior. Due to differences in specific surface area, smaller-diameter HMSs (1000 μm) release drugs more rapidly than larger ones (2000 μm) [25]. This phenomenon can be attributed to the diffusion-driven release mechanism of smaller HMSs, resulting in an extremely fast drug release process [43]. Additionally, microspheres with smaller diameters (≤3 μm) are less likely to clog injection needles, improving injectability. However, excessively small microspheres are prone to phagocytosis by cells [116]. Therefore, selecting an optimal microsphere size is essential to balance small diameter advantages and minimize phagocytosis risk.

In batch emulsion technology, microsphere diameter can be controlled by adjusting the temperature and precursor solution concentration. The most influential factor is the aqueous-to-oil phase ratio; as the aqueous phase proportion increases, the interfacial tension between the two phases decreases, leading to better dispersion and smaller droplet size. Lowering the temperature promotes particle growth, increasing microsphere size [86]. A high stirring rate, on the other hand, induces droplet fragmentation, forming smaller particles [30]. Experimental studies have shown that high concentrations of crosslinking agents inhibit HMS swelling during the reaction, reducing microsphere size [45]. In microfluidic technology, microsphere size and alignment spacing are influenced by the relative pressure of the surfactant at the water/oil interface. This relative pressure determines the size and distribution of counter micelles, where higher relative water pressure facilitates the formation of more complex microsphere arrays [117]. For the spray method, the viscosity of the precursor solution plays a key role in determining microsphere size [118]. Lan et al. demonstrated that a precursor solution with a high PLGA concentration (180 mg/mL) exhibits higher viscosity, resulting in a larger average microsphere size (6 μm) compared to those prepared with a lower PLGA concentration (100 mg/mL), which yielded smaller microspheres (2 μm) [59].

#### 3.1.2. Porosity

The pore structure of HMSs typically consists of porous networks or nanometer-sized pores, offering excellent hydration capacity and a high specific surface area. Increased porosity in HMSs leads to a larger specific surface area, allowing for higher drug loading and more precise controlled release [119]. Conversely, HMSs with lower porosity exhibit reduced water absorption capacity [120]. Therefore, for HMSs used in wound dressings, high porosity is particularly essential [27]. Xu et al. designed high-porosity HMSs capable of rapidly absorbing exudates and releasing drugs to mitigate oxidative stress and prevent bacterial infections during the early stages of wound healing [61]. In drug delivery applications, drug release rates can be finely controlled by precisely designing pore size, morphology, distribution, and porosity. The primary factor influencing porosity is the degree of crosslinking in the microspheres. A higher crosslinking degree results in denser microspheres with fewer pores. This crosslinking degree can be enhanced by increasing the concentration of crosslinking agents or selecting agents with stronger intermolecular interactions. Studies have shown that Schiff base bonds can form between CS microspheres and solid lipid-polymer hybrid nanoparticles (SLPNs) through covalent bonding, significantly strengthening crosslinking and reducing pore space in the microspheres. This structural change prevents SLPN from migrating into the buffer medium [26]. Another study demonstrated that carboxymethyl chitosan (CMCS)-CuO nanoparticles act as an inorganic crosslinking agent with SA, exhibiting strong interfacial interactions. These interactions effectively enhance the crosslinking strength of composite microspheres, thereby reducing the migration paths of water and drugs within HMSs and slowing the release rate of encapsulated drugs [13].

#### 3.1.3. Swelling Degree

Swelling capacity, a critical determinant of drug release behavior from HMSs, is significantly influenced by porosity. Microspheres with higher porosity and open-pore architectures exhibit enhanced water permeability and consequently greater swelling capacity [121]. Notably, increased crosslinking density reduces porosity, leading to diminished swelling responses [109]. The incorporation of charged particles elevates ionic osmotic pressure within microspheres, thereby promoting water absorption and swelling capacity enhancement [108]. Material selection plays a pivotal role, as hydrophilic polymers like SA substantially improve microsphere hydrophilicity and subsequent swelling performance. Due to the abundant hydroxyl groups in layered double hydroxide (LDH) structures, the hydrophilicity of microspheres is enhanced, and the swelling capacity of HMS increases with higher LDH content [34]. Microspheres’ diameter also affects swelling behavior. As the diameter decreases, both swelling rate and capacity increase, promoting drug release [43]. However, excessive swelling can lead to a reduction in HMS polymer concentration, compromising mechanical durability. Therefore, maintaining an optimal swelling degree is crucial [81]. To address this issue, incorporating metal nanoparticles has been proposed as a strategy to regulate HMS swelling behavior [82].

#### 3.1.4. pH-Responsive Drug Release

The HMS-based drug delivery system for antimicrobial purposes is responsive to pH stimuli, capable of undergoing structural changes in specific acidic environments, thereby releasing the drug. For example, acute cutaneous wounds typically have a pH of 4–6, while chronic wounds exhibit a pH of 7–9 [110]. In contrast, degenerative changes in tissue in conditions such as arthritis result in a weakly acidic microenvironment (pH = 6.0). Therefore, HMSs that respond to pH variations in different microenvironments offer strong targeting and selectivity, minimizing side effects on healthy tissues. Some researchers have developed pH-responsive HMSs based on SA, which are particularly suitable for treating chronic wound infections. These microspheres rapidly swell at pH 7.4, triggering the release of antimicrobial drugs due to the effect of pH on the Ca^2+^ and –COO– linkages in the Alg fraction [83]. To prepare pH-responsive HMSs, pH-sensitive materials are typically incorporated into the microspheres. These materials include polycondensates, CS, polydimethyl lactam, poly (methacrylic acid), and PVA [13,122,123]. The primary reaction mechanisms of these pH-sensitive materials include acid-sensitive bond cleavage and protonation of chemical groups [122]. The former refers to the connection of the drug to the microspheres via an acid-sensitive bond, while the latter involves changes in the solubility properties of the microsphere material upon protonation, which facilitates drug release [124].

#### 3.1.5. Photo-Responsive Drug Release

Since endogenous stimulation (e.g., pH) is challenging to monitor and regulate, researchers have developed various exogenous stimulation-responsive HMSs to enable precise drug release under minimally invasive or non-invasive therapeutic conditions. This approach enhances patient compliance and ensures optimal therapeutic outcomes. Among these, exogenous stimulation in antimicrobial drug delivery systems primarily involves photo stimulation, where HMSs are exposed to specific wavelengths of light to induce localized thermal effects or generate reactive oxygen species (ROS). This enhances antimicrobial efficacy while minimizing damage to healthy tissues. Photo stimulation allows for precise control over drug release timing and dosage, offering customizable therapeutic regimens. Photo-sensitive materials play a crucial role in designing photo-responsive microspheres, with common examples including quantum dots, photo-sensitive nanomaterials, and PVA [22,24]. This system integrates the non-invasive nature of light with high spatial resolution, ensuring effective targeting and therapeutic benefits. Photo-responsive microspheres are widely used in bone tissue repair, as they can slightly elevate local temperatures under microwave radiation to trigger drug release, reducing the risk of adverse reactions associated with prolonged exposure to high temperatures [79]. In another study, functionalized gelatin microspheres, coated with polydopamine, demonstrated the ability to generate ROS in response to NIR photo at temperatures ranging from 41 °C to 45 °C. This temperature range is well-tolerated by osteoblast-associated cells, providing a promising approach for sterilization [6].

#### 3.1.6. Magnetic-Responsive Drug Release

In addition to photo stimulation, magnetic stimulation represents a promising development direction due to its insensitivity to ambient photo and its ability to penetrate tissues, thereby offering more stable exogenous control [82]. By incorporating magnetic nanoparticles, primarily iron oxide nanoparticles, into drug delivery systems, magnetic field-induced drug release can be achieved. This method leverages the directionality of the magnetic field to enhance targeting efficiency [53]. One study demonstrated that incorporating Fe_3_O_4_ nanoparticles into drug delivery microspheres resulted in excellent magnetic responsiveness, allowing for rapid movement and aggregation under an external magnetic field, which facilitated localized controlled drug release [78]. Furthermore, magnetic stimulation can be combined with other modalities to create a multimodal therapeutic strategy, thereby enhancing antimicrobial efficacy and expanding the range of applications. This approach is both non-invasive and reproducible, offering a novel solution for refractory infections.

#### 3.1.7. Targeted Release

In response to the differential expression of specific receptors across various diseases, targeting molecules can be designed to modify HMSs. These microspheres are typically modified with ligands (e.g., antibodies, peptides, or glycans) that enable them to recognize specific receptors in lesions, thereby providing new opportunities for precision medicine and personalized therapy. For instance, some researchers have encapsulated the cartilage-targeting peptide WYRGRL in a hydrogel, endowing HMSs with precise cartilage-targeting capabilities [125]. He et al. loaded HMSs with an anti-collagen type I (Anti-Col1) antibody to target early cartilage damage in osteoarthritis (OA), ensuring precise and effective lubrication of early OA lesions [126]. A study also designed an ROS “bomb” targeted at bacterial cell membranes, delivered by HMS. This system consists of hydroxy iron oxide/glucose oxidase/calcium phosphate-coated polydopamine nanoparticles, which can respond to the acidic environment around persistent bacteria and trigger the release of a large amount of ROS to disrupt the cell membrane and kill the bacteria [127]. The antibodies on the surface of HMSs may be captured by immune cells and subsequently cleared by the immune system, making it difficult to achieve long-term drug release. PEGylation is one of the most commonly used immune evasion strategies. By modifying the surface of HMSs with PEG, a hydrophilic protective layer is formed, which can shield the microsphere’s surface antigens and reduce interactions with immune cells. Gheffar et al. performed PEGylation on PLGA microspheres, avoiding unnecessary immune reactions and prolonging the antimicrobial effect [128]. Through targeted delivery, drugs can maximize efficacy in the shortest possible time, optimize treatment regimens, and enhance patient response and quality of life. However, research on the targeted molecular modifications of HMSs remains limited, necessitating further exploration in this area.

### 3.2. Cell Adhesion

HMSs must possess strong adhesion capabilities to ensure stable attachment to the wound surface or to effectively trap bacteria [50]. This adhesion not only facilitates the accumulation of microspheres in localized areas, but also establishes a protective barrier that mitigates the invasion of external stimuli and pathogens, thereby accelerating wound healing and enhancing resistance to infections. The adhesive properties of HMSs can be improved by modifying their surface characteristics, such as incorporating surface coatings or introducing hydrophilic functional groups. Research has demonstrated that the incorporation of polydopamine into microspheres enhances bioadhesion to catecholamines through π-π stacking and hydrogen bonding interactions, ensuring stable adhesion of the microspheres to the wound surface [61]. Additionally, studies have shown that the inclusion of CS as an adhesive in the preparation of microspheres results in products with excellent properties [27].

### 3.3. Biocompatibility

The biocompatibility of microspheres is of paramount importance, as it directly influences their safety and efficacy in living organisms. Poor biocompatibility may trigger immune reactions, tissue inflammation, or cytotoxicity. Inflammation alters the local pH and vascular permeability, affecting drug release and distribution. Immune responses can cause mild symptoms such as rashes and itching, or severe symptoms like difficulty breathing and shock, which limit their clinical translation. Cytotoxicity may inhibit cell proliferation, affect tissue repair, or cause membrane damage, leading to leakage of cellular contents [38]. Therefore, researchers should aim to prepare HMSs using materials demonstrated to possess high biocompatibility, ensuring compatibility with the biological environment. This approach can reduce adverse reactions and enhance therapeutic efficacy. In one study, researchers assessed the biocompatibility of C-AMP using Chinese hamster ovary cells, finding that over 80% of the cells remained viable in the presence of C-AMP [31]. Direct delivery of metal ions into the body may lead to toxic reactions, necessitating careful consideration of the biocompatibility of metal nanoparticles when delivered via HMSs. Some researchers tested the toxicity of microspheres loaded with AgNPs compared to those loaded with polydopamine using bone marrow mesenchymal stem cells, and the results indicated that the release of both types at lactate dehydrogenase levels was similar, suggesting that the released silver ions do not compromise cell membrane integrity [76].

Hemolytic activity is a crucial indicator for evaluating the biocompatibility of HMSs. High hemolytic activity can destroy red blood cells, leading to hemolysis, which in turn causes immune reactions and toxicity, posing a significant safety hazard for drug delivery systems and biomaterial applications. Therefore, researchers should prepare HMSs using materials that have been shown to exhibit low hemolytic activity, as this property is essential for their application in clinical and biomedical fields. By regulating properties of HMSs—such as surface modification, particle size control, and charge optimization—their hemolytic activity can be significantly reduced, thereby minimizing interactions with erythrocytes and reducing blood damage. In one study, dimethyldiallylammonium chloride was incorporated into HMSs, which not only endowed the microspheres with the ability to attract bacteria through charge, but also reduced hemolytic activity; notably, there was no significant difference in the number of hemocytes compared to control samples after blood culture [62]. In another study, cellulose-based HMSs induced only 2% erythrocyte lysis even at high concentrations, demonstrating their low hemolytic activity [61].

### 3.4. Degradability

HMSs degrade after drug release to minimize their damage and side effects on the human body. The degradation products are typically small biodegradable molecules or harmless metabolites that can be eliminated through the body’s natural metabolic pathways, thus avoiding the potential harm associated with long-term retention. This property provides HMSs with a significant advantage in clinical applications, particularly in the long-term use of drug delivery systems, and substantially reduces the burden of accumulation on the body. By regulating the degradation rate of HMSs, precise control over drug release can be achieved. Rapidly degrading HMSs can be cleared quickly, making them suitable for short-term treatments, while slowly degrading HMSs can continuously release drugs, ensuring sustained therapeutic effects. However, an excessively long degradation time may lead to cytotoxicity and limit their applications; therefore, the degradation rate should be adjusted within an appropriate range [54]. One study demonstrated that CMS/CS microspheres could degrade into water and carbon dioxide in the presence of lysozyme, achieving a degradation rate of approximately 68.7% after three days, highlighting their potential as a biodegradable drug delivery system [26]. Additionally, Jin et al. prepared HMS patches that could completely degrade three days after application to mouse wounds, thereby eliminating the need for surgical removal of HMS patches during treatment [105].

### 3.5. Mechanical Properties

The mechanical properties of microspheres must be sufficiently strong to provide adequate support, preventing their breakage, which could lead to a burst release of the drug and reduce its efficacy. For instance, in bone defect repair, both HMSs and scaffolds require strong mechanical properties to support the wound until new bone is formed [79]. Microspheres with poor mechanical properties are prone to deformation and can easily clog the needle during injection, making the administration difficult [129]. Additionally, HMSs must maintain high mechanical strength under elevated temperature conditions to prevent softening, rupture, or deformation, which is particularly critical in applications that involve mechanical stress. The mechanical properties and thermal stability of microspheres can be enhanced by incorporating metal nanoparticles, bioactive materials, or by utilizing crosslinking between different materials [78,130]. Chen et al. demonstrated that the introduction of CaCO_3_ particles into Alg microspheres, facilitated by the strong interactions between CaCO_3_ and Alg molecules, formed a robust internal framework, significantly enhancing the mechanical properties [49]. While pure SA microspheres fragmented at a pressure of 5 MPa, the SA/cellulose nanocrystal (CNC) hybrid microspheres remained structurally intact at a pressure of 20 MPa, indicating that the addition of cellulose nanocrystals effectively improved the mechanical properties of the material [15]. Furthermore, the mechanical properties are closely related to the degradation rate of microspheres, and achieve a reasonable balance between the two ensuring the stability and controlled degradation characteristics of microspheres in vivo.

Typically, the mechanical strength of hydrogels is influenced by their hydration state, crosslinking density, and material composition, which also affect their thermal stability. Wang et al. found that pure CS microspheres exhibit a hemispherical shape in the dry state, while SA/cellulose nanocrystal (SLPN-CS) nanocomposite microspheres maintain their original spherical shape without significant surface collapse, indicating that their thermal stability is significantly enhanced following functionalization [26]. Additionally, it was demonstrated that hemostatic porous microspheres self-assembled from SA/CNC and the antimicrobial polymer EPL exhibited greater thermal stability than both SA microspheres and SA/CNC microspheres, as evidenced by a lower percentage mass loss at the same temperature [15].

## 4. Application of Drug-Loaded Antimicrobial Hydrogel Microspheres (HMSs)

**Figure 7:**
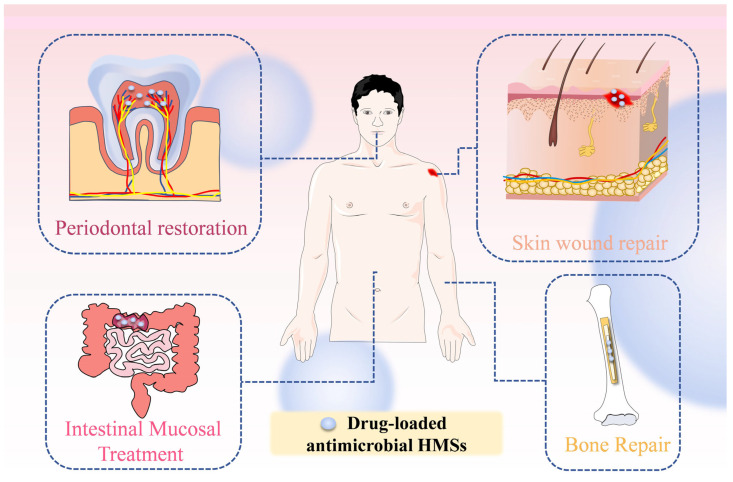
Application of drug-loaded antimicrobial HMSs in vivo. Periodontal restoration, involving localized delivery of antimicrobial agents and growth factors to suppress pathogens and promote periodontal tissue regeneration; Skin wound repair, leveraging the hydrogel’s moist environment and drug-loading capacity to accelerate healing while preventing infections; Intestinal Mucosal Treatment, enabling targeted drug delivery to intestinal lesions for precise anti-inflammatory or mucosal repair effects; Bone repair, integrating osteoinductive components with sustained drug release to synergistically enhance osteogenesis and anti-infection capabilities in bone defect regions.

The emergence of HMSs has addressed the deficiencies of traditional drug delivery systems. These conventional systems exhibit several shortcomings: (1) they may lead to rapid drug release, causing concentrations to fall below therapeutic levels, resulting in wastage of both the drug and materials; (2) they are not able to deliver drugs to more intricate tissue structures; (3) drugs can be degraded by gastrointestinal enzymes, rendering them ineffective; (4) they may exhibit cytotoxicity. As drug carriers, HMSs show great promise in applications such as skin wound dressings and bone repair. Their primary advantage lies in overcoming the limitations of traditional therapies by establishing a dynamic repair system that integrates infection control, inflammation modulation, and tissue regeneration. Due to their benefits in controlled drug release, tissue targeting, penetration, and injectability, HMSs are commonly utilized in minimally invasive drug delivery. Furthermore, the high water content and low cytotoxicity of HMSs enable them to mimic the extracellular matrix, creating an ideal environment for wound healing. Their high porosity also facilitates cell adhesion and the transport of nutrients and metabolic waste, effectively alleviating inflammation. The common application directions of drug-loaded antimicrobial HMSs are summarized (Figure 7).

### 4.1. Skin Wound Repair

HMSs have revolutionized traditional dressings in wound treatment by transcending the passive barrier function and achieving dynamic repair through a triadic synergistic mechanism of “microenvironment remodeling, antimicrobial action, and healing promotion,” particularly in the context of infected wounds [131]. They maintain a moist microenvironment around the wound, thereby preventing secondary damage. Various antimicrobials are often co-delivered with growth factors such as vascular endothelial growth factor (VEGF) and bone morphogenetic protein-2 (BMP-2) to enhance angiogenesis and tissue regeneration [46]. For instance, composite HMSs constructed with zinc ions (Zn^2+^) and histidine-tagged VEGF (His-VEGF) exhibit dual effects of antimicrobial action and enhanced angiogenesis, while also facilitating the remodeling of the wound microenvironment [110]. Recent research trends have concentrated on developing multifunctional systems that integrate “antimicrobial, antioxidant, and healing promotion” properties. For example, safflower polysaccharide-HMS hydrogels disrupt the vicious cycle of inflammation by scavenging ROS, providing new insights for treating challenging wounds, such as diabetic foot ulcers [107].

### 4.2. Intestinal Mucosal Treatment

The pathogenesis of inflammatory bowel diseases (IBDs) is primarily driven by dysregulated inflammatory responses to intestinal microbiota in genetically predisposed individuals, with environmental factors acting as potential triggers [132]. Current therapeutic strategies for IBD frequently employ antimicrobial drug delivery systems. Owing to the complexity of IBD pathology, HMSs offer distinct mucosal targeting advantages. These advantages can be amplified through charge modification or pH-responsive materials to achieve site-specific delivery. For instance, rectally administered dopamine-functionalized anionic HMSs demonstrated robust adhesion to the cationic mucosal surfaces in inflamed tissues, effectively scavenging ROS while releasing Zn^2+^ and mesalazine. This dual action exhibited potent antimicrobial effects and markedly alleviated acute colitis in experimental models [41]. Similarly, CMS/CS-coated HMSs responded to colonic pH by releasing citric acid-derived graphene quantum dots (GQDs) and naproxen, achieving antimicrobial efficacy without cytotoxic effects [26]. Strategic coating modifications further enhance HMS stability in intestinal environments. For example, Yuan et al. utilized chitosan-coated HMSs for ciprofloxacin delivery, significantly improving both drug stability and therapeutic outcomes [96]. In another approach, potassium dimethanolate and P-type zeolite molecular sieves encapsulated within SA/konjac glucomannan/CS composite HMSs demonstrated broad-spectrum antimicrobial activity against enteric pathogens. The incorporation of zeolite P notably enhanced system stability under simulated intestinal conditions, positioning this formulation as a viable non-antibiotic bacterial inhibitor for veterinary gastrointestinal applications [14].

### 4.3. Tissue Repair

#### 4.3.1. Bone Repair

HMSs have emerged as a promising platform for bone defect repair owing to their precisely tunable dimensions, minimally invasive delivery capability, and efficient encapsulation of bioactive factors or therapeutic agents. Research indicates that HMSs within an optimal diameter range (<200 μm) significantly enhance bone regeneration, as demonstrated in the in vivo studies [133]. Addressing infectious bone defects necessitates a multifunctional strategy that concurrently achieves antimicrobial efficacy, osteogenesis, and angiogenesis. For instance, Dai et al. engineered dual-crosslinked HMSs incorporating Cu^2+^ ions for potent antimicrobial effects, synergistically combined with VEGF and BMP-2 to drive vascularization and osteoregeneration [52]. Complementary studies on copper-doped mesoporous bioactive glass (Cu-MBG) revealed sustained Cu^2+^ release, which simultaneously stimulates osteogenic differentiation, angiogenesis, and microbial inhibition. This dual-functional system enables localized, controlled drug delivery for enhanced bone regeneration and infection management [98]. Hydroxyapatite, mimicking the inorganic composition of native bone, has been extensively utilized in bone tissue engineering due to its inherent osteoconductive properties and capacity to induce osteogenic differentiation [134]. Innovative hydroxyapatite/gelatin HMSs co-loaded with tetracycline TH and AgSD demonstrated dual functionality in antimicrobial defense and bone matrix deposition [78]. Furthermore, porous hydroxyapatite microspheres achieved sustained AMX release during defect remodeling, effectively resolving infected bone defects [69]. For recalcitrant conditions such as MRSA-induced chronic osteomyelitis, complete osseous restoration remains the therapeutic endpoint. A breakthrough core-shell composite material addresses this challenge through a tri-modal mechanism: a magnesium phosphate cement scaffold provides structural support, VAN-loaded HMSs ensure prolonged antimicrobial action, and Fe_3_O_4_-functionalized nanosheets enhance mechanical stability. This system achieved an unprecedented 99.98% MRSA eradication rate while significantly accelerating bone repair in preclinical models [79].

#### 4.3.2. Periodontal Repair

Inflammatory bone loss in tooth-supporting tissues, frequently caused by bacterial infections, necessitates therapeutic strategies that enable precise spatiotemporal control of drug release tailored to individual patient profiles. HMSs have demonstrated significant potential in achieving personalized periodontal therapy through programmable drug delivery systems. For example, researchers engineered a dual-drug hydrogel system targeting *P. gingivalis* infections, where minocycline and clindamycin were encapsulated into the acidic and esterified termini of PLGA microspheres, respectively. By modulating the acid-to-ester ratio in PLGA formulations, this system achieved tunable release kinetics, thereby enabling patient-specific treatment regimens [60]. The sustained-release capabilities of HMSs critically extend therapeutic duration while minimizing dosing frequency, substantially improving patient adherence. Mou et al. developed serum albumin-based HMSs co-loaded with minocycline and zinc oxide nanoparticles, which maintained controlled drug release over 72 h. Compared to conventional 2% minocycline ointments, this innovative system not only prolonged antimicrobial efficacy, but also significantly enhanced gingival tissue regeneration capacity, as evidenced by histological analyses [16].

### 4.4. Other Applications

In addition to the previously mentioned applications, drug-loaded antimicrobial HMSs are utilized across various fields. In cancer therapy, CS microspheres loaded with 5-fluorouracil (5-FU) and tetracycline hydrochloride have been shown to inhibit the proliferation of HepG2 cells while also exhibiting significant antimicrobial effects against *E. coli* and *S. aureus* [28]. Another innovative application lies in blood purification. Microspheres prepared by combining amphoteric sulfoxymethyl methacrylate betaine, dimethyldiallylammonium chloride, and rabbit erythrocyte membranes act as blood perfusion adsorbents. These HMSs attract bacteria and adsorb bacterial toxins, enabling blood purification and providing a treatment option for sepsis caused by Gram-positive bacteria [62]. Additionally, antimicrobial HMS adsorbents have been applied to remove Mg^2+^, Ca^2+^, and bacteria from drinking water, effectively softening hard water. These HMSs are reusable, enhancing their practicality and sustainability [135].

## 5. Summary and Outlook

In summary, HMSs demonstrate significant potential as drug carriers for delivering antimicrobials. They effectively address the limitations of traditional drug carriers, such as high cost, low biocompatibility, low drug-loading capacity, and high cytotoxicity. HMSs can deliver a variety of antimicrobials, including antibiotics, antimicrobial peptides, and metal nanoparticles. Numerous studies have confirmed their excellent antimicrobial effects, providing a promising reference for the future development of antimicrobial drug delivery systems.

This review highlights the synergistic effects of HMSs combined with secondary hydrogels, as well as multifunctional microspheres prepared from composite materials. During HMS production, characteristics such as diameter, porosity, and mechanical strength significantly influence their performance and specific applications. To guide future research, a comprehensive database of HMSs performance parameters should be established, encompassing key data such as solubility, porosity, mechanical strength, and biocompatibility. Currently, the application of antimicrobial HMSs is primarily focused on wound dressings and bone repair, with limited attention given to other fields like water purification, agricultural biocides, food preservation, and antimicrobial textiles. Expanding HMS applications could lead to innovative solutions, such as developing microspheres that combine crop growth promotion with antimicrobial properties. Such advancements would improve pesticide efficiency, reduce environmental pollution, and enhance crop yields.

Although a variety of HMSs for delivering antimicrobials have been fabricated, their further application still faces significant challenges. The main techniques currently used to produce HMSs all have distinct advantages and limitations. A potential solution lies in developing hybrid processes that combine the strengths of these methods. For instance, microfluidics could be used to control microsphere homogeneity, followed by spraying to enable large-scale production. This approach could yield homogeneous HMSs at higher production rates, presenting a promising direction for future HMS fabrication. Regulating key HMS properties, such as porosity and degradability, remains difficult with current preparation techniques. While HMSs exhibit good biocompatibility, the antimicrobials they carry or their metabolites may pose cytotoxic risks. Careful drug selection is essential to minimize toxicity. Similarly, regulating the drug release process is critical; a release that is very rapid can lead to cytotoxic accumulation, while an overly slow release may fail to achieve the minimum inhibitory concentration, potentially promoting bacterial resistance. Another limitation is the narrow range of bacterial species used to evaluate the antimicrobial performance of drug-loaded HMSs, with most studies focusing on *S. aureus* and *E. coli*. Expanding testing to a broader range of bacterial species will be important for widening their application scope. Furthermore, as single-function HMSs are insufficient to meet increasingly complex market demands, future research should focus on the design and optimization of multifunctional HMSs to address diverse and individualized needs.

Currently, there are several potential obstacles to obtaining regulatory approval for the delivery of antimicrobials using HMSs, primarily related to safety, efficacy, and manufacturing quality control. Regarding safety, producers must use FDA-approved materials for microsphere preparation and demonstrate that the degradation products of HMSs will not cause adverse reactions in the body. In terms of efficacy, the antimicrobial spectrum of HMSs delivering antimicrobials must be clearly defined both in vitro and in vivo, and attention should be given to the issue of bacterial resistance. Moreover, manufacturing processes should be optimized, and strict quality standards should be established to control the production quality of HMSs. HMSs with excellent performance can address many issues present in traditional carriers. When conducting clinical antimicrobial trials with HMSs, close collaboration with clinicians is essential to understand clinical needs and pain points, selecting indications for resistant bacteria or those with suboptimal responses to existing therapies. Comprehensive preclinical safety and efficacy evaluations should be conducted, and clinical trial protocols should be carefully designed with clear clinical endpoints (e.g., infection clearance rates, symptom relief time). However, clinical trials also face challenges such as regulatory approval, funding support, and patient recruitment, necessitating thorough preparation. The anticipated commercialization timeline for HMSs in antimicrobial delivery could be 10–15 years, influenced by clinical trial progress, regulatory approval speed, and funding availability. To accelerate commercialization, strategies such as utilizing fast-track regulatory pathways, early interactions with regulatory agencies, and leveraging existing data may be considered.

## Figures and Tables

**Table 1:** Comparison of HMS with conventional carriers.

Carrier Type	Advantages	Disadvantages
HMSs	High biocompatibility; Ability to penetrate deep tissues; Controlled release; High drug loading	The production process needs to be optimized; Complex preparation process
Natural polymer hydrogels	High biocompatibility; Low cost	Difficulty in achieving deep tissue drug delivery; Low mechanical strength
Synthetic polymer hydrogels	Adjustable mechanical strength; Functional design flexibility; High drug-loading potential	Low biocompatibility; Complex preparation process
Liposomes	High biocompatibility; Hydrophobic drugs can be encapsulated	Low drug loading; Poor stability; Prone to leakage
Dendritic polymers	High drug-loading potential	High cytotoxicity; High production cost; Slow degradation
Free antimicrobials	Easy to use; No carrier preparation costs	Difficult to accurately control release; Side effects; Poor stability

**Table 2:** The advantages and disadvantages of HMSs preparation technology as well as antimicrobial application and preparation materials.

Preparation Methods	Materials	Advantages of Preparation Method	Disadvantages of Preparation Method	Antimicrobial Application
Batch emulsion	Alg [14,24], Alg/PLGA [25], CS [17,26,27,28,29], CMCS [30], Low molecular weight CS [31], HA [32,33], CMC [34], CS/CMC [35], CS/Alg [36], CS/Carboxymethyl starch (CMS) [37], CS/κ-Carrageenan [38], Gelatin [39], Debranched starch [40], GelMA [32], Konjac glucomannan [14,41], Poly(hydroxybutyrate-co-hydroxyvalerate) [42], PLGA [43,44,45,46,47], PVA [45,48]	Simple preparation process High yield Low cost	Waste generation Polydispersity of microspheres Batch-to-batch variations Uneven drug loading Cumbersome purification steps	Large-scale production Non-uniform drug loading/size acceptable
Microfluidic	Alg [49,50], GelMA [51], Alg/GelMA [52], CS [53], CS/Alg [54], Gelatin [22], Polyacrylamide(pAAm) [55]	Precise control over production process Highly homogeneous particle size Even drug loading Low batch-to-batch variations	Relative expensive equipment cost Low yield	Precise size/release control Continuous release and synergy preferred
Spraying	Alg [56,57], GelMA [58], PLGA [59,60], Cellulose [61], Sulfoxylbetaine methacrylate [62]	Relative high yield Low pollution Preparation of high-purity microspheres	Inadequate regulation of particle size in HMSs Relatively high initial investment and learning cost	High drug stability/activity Size uniformity less critical
Photolithography	Polyethylene glycol (PEG)/polyaniline [63], PVA [64], Gelatin [65]	High resolution Highly homogeneous particle size Easy size adjustment Low pollution	High cost Low yield	Photo-responsive applications Shape-controlled drug regulation
3D printing	Alg [66], GelMA/CS [67], PEG [68], Collagen/SA [69]	Precise control of internal structure Personalized customization	Slow print High cost	Complex 3D structures Personalized HMSs preparation
Phase Separation	Alg [70], poly-(N-isopropylacrylamide) [71], CS [72]	Simple preparation process Wide range of application High yield	Solvent residue High pollution Poorly homogeneous particle size	Contamination control prioritized Size uniformity non-essential

**Table 3:** Application and antimicrobial activity of HMSs for the delivery of antimicrobials.

Applications	Loaded Antimicrobials	Antimicrobial Activities	Ref.
Skin wound repair	TH, AgSD	*S. aureus*, *E. coli*	[73]
	*L. reuteri*	*S. aureus*, *E. coli*, *S. enterica*	[32]
	Rifamycin	*S. aureus*	[49]
	AgNPs, gentamicin	*S. aureus*, *E. coli*	[33]
	Sanguinarine	MRSA, *E. coli*	[39]
	AgNPs	*S. aureus*, *E. coli*	[56]
	Zn^2+^	*S. aureus*, *E. coli*	[20]
	Gallium maltolate	MRSA	[59]
	Lysozyme, MXene	*S. aureus*	[24]
	TH	*S. aureus*, *E. coli*	[30]
	Rifampin, streptomycin	*M. ulcerans*	[42]
	PHMB	*S. aureus*, *E. coli*, *P. aeruginosa*	[46]
	Zn^2+^	*S. aureus*, *E. coli*	[53]
	EPL	*S. aureus*, *E. coli*, *P. aeruginosa*	[15]
	VAN, gentamicin	MRSA, *E. coli*, *P. aeruginosa*	[48]
	Antimicrobial peptide	*E. coli*	[22]
	Thymol	*S. aureus*, *E. coli*	[61]
	Zeolitic imidazolate framework-8 nanoparticles	*S. aureus*, *E. coli*	[25]
	Ciprofloxacin	MSSA, MRSA	[47]
	*Melaleuca alternifolia* oil	*B. cereus*, *S. aureus*, *E. coli*, *S. enterica*	[36]
	EPL	*S. aureus*, *E. coli*	[74]
	EGCG	*S. aureus*, *E. coli*, *S. typhi*	[40]
	*L. reuteri*	*S. aureus*, *E. coli*	[50]
	AgNPs	*S. aureus*, *E. coli*	[75]
Bone repair	AMX	*E. coli*	[69]
	AgNPs	*S. aureus*	[76]
	Ag-hydroxyapatite	*S. aureus*, *E. coli*	[58]
	Cu^2+^	*S. aureus*, *E. coli*	[52]
	LL-37	*E. coli*	[45]
	VAN	*S. aureus*	[77]
	TH, AgSD	*S. aureus*, *E. coli*	[78]
	VAN	MRSA	[79]
	VAN, total flavonoids of Rhizoma Drynariae	*S. aureus*, *E. coli*	[29]
Intestinal mucosal treatment	AMX	*S. aureus*, *E. coli*	[34]
	K-diformate	*S. aureus*, *E. coli*, *B. subtilis*	[14]
	Olazazine, Zn^2+^	*E. coli*	[41]
	GQDs	*S. aureus*, *E. coli*	[26]
Periodontal restoration	*C. reinhardtii*	*S. gordonii*, *F. nucleatum*, *P. gingivalis*	[80]
	VAN	*S. aureus*	[44]
	Doxycycline, lipoxin	*P. gingivalis*	[60]
	Amino antibacterial nanoparticles	*P. gingivalis*	[6]
Agricultural sterilization	Citral	*S. aureus*, *E. coli*, *B. subtilis*, *B. cinerea*	[35]
Water purification	AgNPs	*S. aureus*, *E. coli*	[81]
	AgNPs	*S. aureus*, *B. subtilis*, *E. coli*, *P. aeruginosa*	[45]
Food preservation	Mangiferin	*V. parahaemolyticus*, *V. cholerae*	[38]
Anti-infective therapy	Si@Ni	*P. aeruginosa*, *K. pneumoniae*, MRSA	[51]
	AMP	*C. tropicalis*, *S. cerevisiae*, *C. parapsilosis*	[31]
	AgNPs, Fe_3_O_4_	*S. aureus*, *E. coli*	[82]
	AgNPs	*S. aureus*, *St. thoraltensis*, *P. vulgaris*, *K. pneumoniae*, *E. coli*, *P. aeruginosa*	[17]
	Cefixime	*S. typhi*, *B. subtitles*	[83]
	HRP, IAA	*S. aureus*	[55]
	Bacteriophage, meropenem	*P. aeruginosa*	[57]
Anti-cancer	5-FU, TH	*S. aureus*, *E. coli*	[28]
Sinusitis	Dexamethasone	*S. aureus*, *E. coli*	[43]

*S. gordonii*, *Streptococcus gordonii*; *F. nucleatum*, *Fusobacterium nucleatum*; *P. gingivalis*, *Porphyromonas gingivalis*; *S. enterica*, *Salmonella enterica*; MSSA, Methicillin-Sensitive *Staphylococcus aureus*; *K. pneumoniae*, *Klebsiella pneumoniae*; *M. ulcerans*, *Mycobacterium ulcerans*.

## Abbreviations

The following abbreviations are used in this manuscript:

HMSs	Hydrogel microspheres
PBS	Phosphate-buffered saline
PDMS	Polydimethylsiloxane
Alg	Alginate
CS	Chitosan
MMP-9	Matrix metalloproteinase-9
GelMA	Gelatin methacrylate
HA	Hyaluronic acid
CMC	Carboxymethyl cellulose
PLGA	Poly (lactic acid-hydroxyacetic acid)
LA	Lactic acid
GA	Glycolic acid
PVA	Polyvinyl alcohol
KGM	Konjac glucomannan
SA	Sodium alginate
GO	Graphene oxide
AMP	Amphotericin B
C-AMPs	CS microspheres encapsulating amphotericin B
LDH	Layered double hydroxide
AMX	Amoxicillin
*S. typhi*	*Salmonella typhi*
*B. subtilis*	*Bacillus subtilis*
*S. aureus*	*Staphylococcus aureus*
*E. coli*	*Escherichia coli*
rhBMP-2	Recombinant human bone morphogenetic protein-2
VAN	Vancomycin
TH	Tetracycline hydrochloride
AgSD	Silver sulfadiazine
HAp	Hydroxyapatite
MHGMs	Magnetic hydroxyapatite/gelatin microspheres
ATP	Adenosine triphosphate
AgNPs	Silver nanoparticles
PAA	Poly (acrylic acid)
EGCG	Epigallocatechin gallate
*S. typhimurium*	*Salmonella typhimurium*
*V. parahaemolyticus*	*Vibrio parahaemolyticus*
*V. cholerae*	*Vibrio cholerae*
MIC	Minimum inhibitory concentration
PHMB	Polyhexamethylene biguanide
W-Gels	Double-crosslinked nanocomposite gels
*L. reuteri*	*Lactobacillus reuteri*
*P. aeruginosa*	*Pseudomonas aeruginosa*
PEG	Polyethylene glycol
pAAm	Polyacrylamide
CMS	Carboxymethyl starch
SLPN	Solid lipid-polymer hybrid nanoparticles
CMCS	Carboxymethyl chitosan
ROS	Reactive oxygen species
NIR	Near-infrared
OA	Osteoarthritis
CNC	Cellulose nanocrystals
SLPN-CS	SA/cellulose nanocrystal
BMP-2	Bone morphogenetic protein-2
VEGF	Vascular endothelial growth factor
Zn^2+^	Zinc ions
His-VEGF	Histidine-tagged VEGF
IBD	Inflammatory bowel diseases
GQDs	Graphene quantum dots
Cu-MBGs	Copper-doped mesoporous bioactive glass
5-FU	5-fluorouracil
*S. gordonii*	*Streptococcus gordonii*
*F. nucleatum*	*Fusobacterium nucleatum*
*P. gingivalis*	*Porphyromonas gingivalis*
*S. enterica*	*Salmonella enterica*
MSSA	Methicillin-Sensitive *Staphylococcus aureus*
*K. pneumoniae*	*Klebsiella pneumoniae*
*M. ulcerans*	*Mycobacterium ulcerans*
